# Case Report: Urachal abscess complicated by interloop abscess

**DOI:** 10.3389/fmed.2026.1972646

**Published:** 2026-10-29

**Authors:** Xue-Wu Liu, Tao Luo, Jun-Qiang Liang, Nan Lei

**Affiliations:** Department of Radiology, The People’s Hospital of Lezhi, Lezhi, China

**Keywords:** case report, computed tomography, interloop abscess, magnetic resonance imaging, urachal abscess

## Abstract

Urachal abscess, a rare infection arising from a congenital urachal remnant, is uncommonly complicated by secondary interloop abscess. We report a case of urachal abscess complicated by interloop abscess in an elderly woman. The patient presented primarily with lower abdominal pain and a palpable suprapubic mass, with mildly elevated serum levels of carbohydrate antigen 125 (CA125) and carcinoembryonic antigen (CEA). Abdominal computed tomography (CT) and magnetic resonance imaging (MRI) revealed two adjacent thick-walled cystic lesions, one in the space of Retzius and the other posterior to the first lesion. Both lesions showed peripheral rim enhancement, enhancing internal septations, non-enhancing fluid components, and marked hyperintensity on diffusion-weighted imaging (DWI). Thin-slice contrast-enhanced CT clearly depicted a tract-like communication connecting the two lesions. Operative findings confirmed that the lesion in the space of Retzius originated from the urachus, whereas the posterior lesion arose from the interloop space. Histopathological examination showed suppurative inflammation with abscess formation in the urachal lesion. We report the clinical features and imaging findings of this case to raise clinicians’ awareness of this rare complication and to offer preliminary imaging clues that may assist in its preoperative recognition.

## Background

The urachus, an embryonic remnant of the allantois, normally undergoes obliteration and fibrosis before birth to form the median umbilical ligament (1). Incomplete obliteration may result in various congenital anomalies, including urachal cyst, urachal diverticulum, urachal sinus, and patent urachus (2, 3). Among adult patients with urachal remnant anomalies, urachal cyst is the most common. The cyst is usually asymptomatic; however, secondary infection may lead to urachal abscess, which typically presents with a midline lower abdominal mass, abdominal pain, fever, and irritative bladder symptoms (4, 5). The infection is generally confined to the extraperitoneal prevesical space (space of Retzius). Interloop abscess, a subtype of intra-abdominal abscess, most commonly occurs secondary to postoperative infection, inflammatory bowel disease, or perforation of a hollow viscus (6). A urachal abscess acting as the primary focus and giving rise to a secondary interloop abscess is exceedingly rare. Here, we report a case of urachal abscess complicated by an interloop abscess and retrospectively analyze its clinical features and imaging findings to enhance clinicians' awareness of this condition.

## Case report

A 61-year-old woman was admitted with a 20-day history of paroxysmal lower abdominal pain and no identifiable trigger. She denied abdominal distension, diarrhea, tenesmus, or irritative bladder symptoms (urinary frequency, urgency, and dysuria), fever, or chills. Physical examination revealed a 7×7 cm lower abdominal mass with no obvious tenderness and limited mobility. No purulent umbilical discharge was observed. Her surgical history included a subtotal abdominal hysterectomy 20 years earlier, and she had a history of diabetes for more than 10 years. Admission laboratory tests showed a white blood cell count of 6.04×10^9^/L (reference range, 3.5–9.5×10^9^/L), urine leukocytes (++), Glycated Hemoglobin (HbA1c) of 11.54% (reference range, 4%–6.1%), carcinoembryonic antigen (CEA) of 5.90 ng/mL (reference range, 0–5 ng/mL), and carbohydrate antigen 125 (CA125) of 41.909 U/mL (reference range, 0–37.22 U/mL).

On admission, non-contrast and contrast-enhanced computed tomography (CT) of the lower abdomen and pelvis were performed. CT revealed two adjacent thick-walled cystic lesions, one in the space of Retzius and the other posterior to the first lesion, measuring 4.9×4.4 cm and 7.7×2.7 cm, respectively. Both lesions had ill-defined margins, with hazy linear stranding of the surrounding perilesional fat (Figure 1A). The anterior lesion was located within the Retzius space, and the posterior lesion was surrounded and wrapped by adjacent small bowel loops. On contrast-enhanced images, both lesions showed marked peripheral rim enhancement, non-enhancing fluid content, and prominent enhancing internal septations. Furthermore, the intestinal wall adjacent to the interloop abscess is thickened, and exudative lesions can be observed in some of the mesenteric areas (Figure 1B-F). The lesion in the space of Retzius communicated with the posterior interloop lesion through a tract-like communication (Figure 1C).

**Figure 1:**
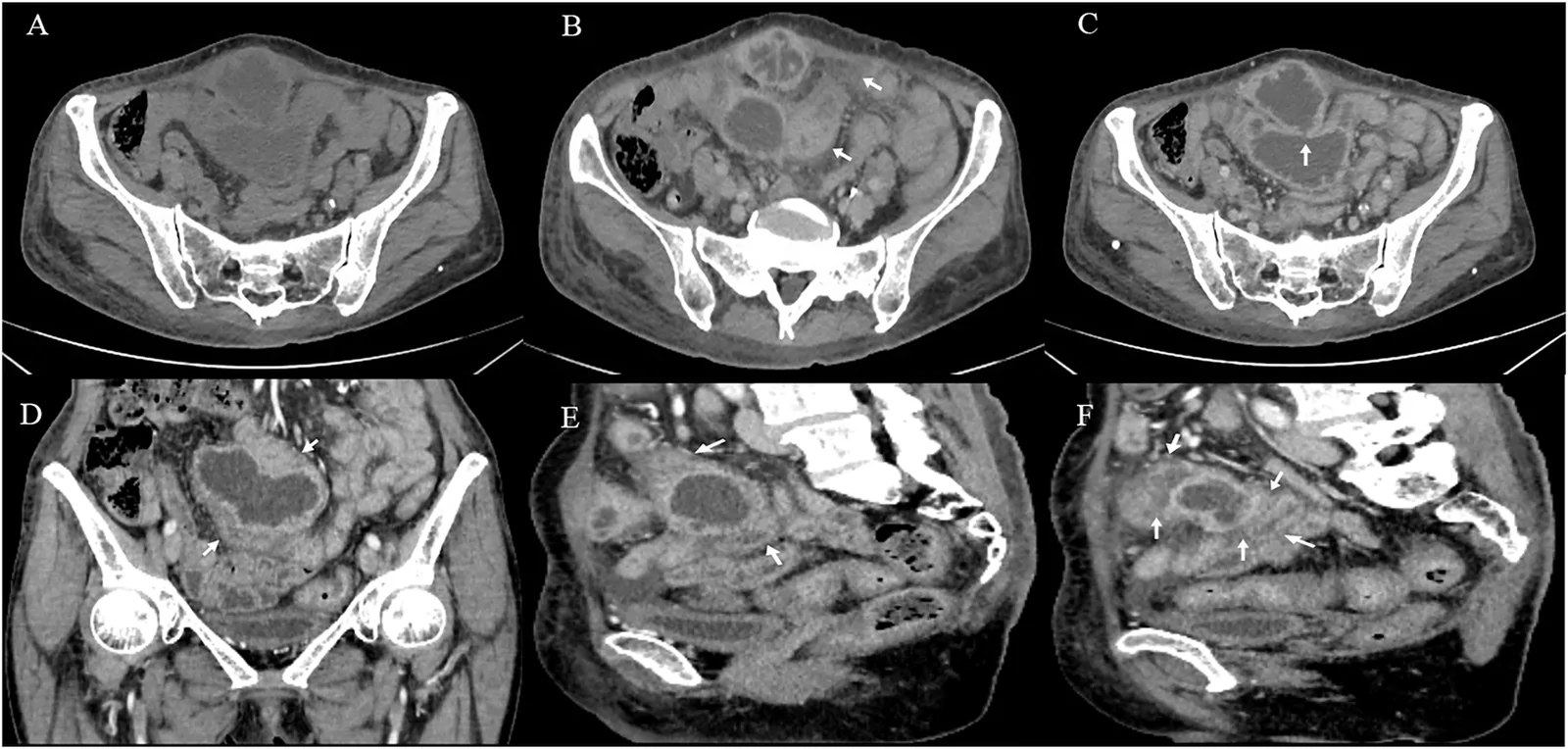
**(A)**: non-contrast CT on the day of admission. **(B–F)**: Axial, coronal, and sagittal contrast-enhanced CT on the day of admission. The lesion in the anterior compartment was located within the space of Retzius. On the sagittal and coronal images, the posterior abscess cavity was surrounded and wrapped by adjacent small intestinal loops (D-F, arrow). The lesion in the space of Retzius communicated with the posterior interloop lesion through a tract-like communication (C, arrow). Both lesions showed marked peripheral rim enhancement, non-enhancing fluid content, and prominent enhancing internal septations. There was some thickening of the intestinal wall around the posterior abscess cavity, and inflammatory exudate can be seen in the mesentery (B, arrow).

Contrast-enhanced pelvic magnetic resonance imaging (MRI) was performed the following day. Non-contrast MRI revealed two adjacent thick-walled cystic lesions, one in the space of Retzius and the other posterior to the first lesion, measuring approximately 4.8×4.0 cm and 6.1×3.6 cm, respectively (Figure 2A). Both lesions were hyperintense on fat-suppressed T2-weighted imaging and diffusion-weighted imaging (DWI), with corresponding hypointensity on the apparent diffusion coefficient (ADC) map (Figures 2B,C). On fat-suppressed contrast-enhanced T1-weighted imaging, both lesions showed marked peripheral rim enhancement, non-enhancing fluid content, and prominent enhancing internal septations (Figure 2D). In addition, sagittal fat-suppressed contrast-enhanced T1-weighted images demonstrated continuity between the bladder dome and the space of Retzius lesion (Figure 2E).

**Figure 2:**
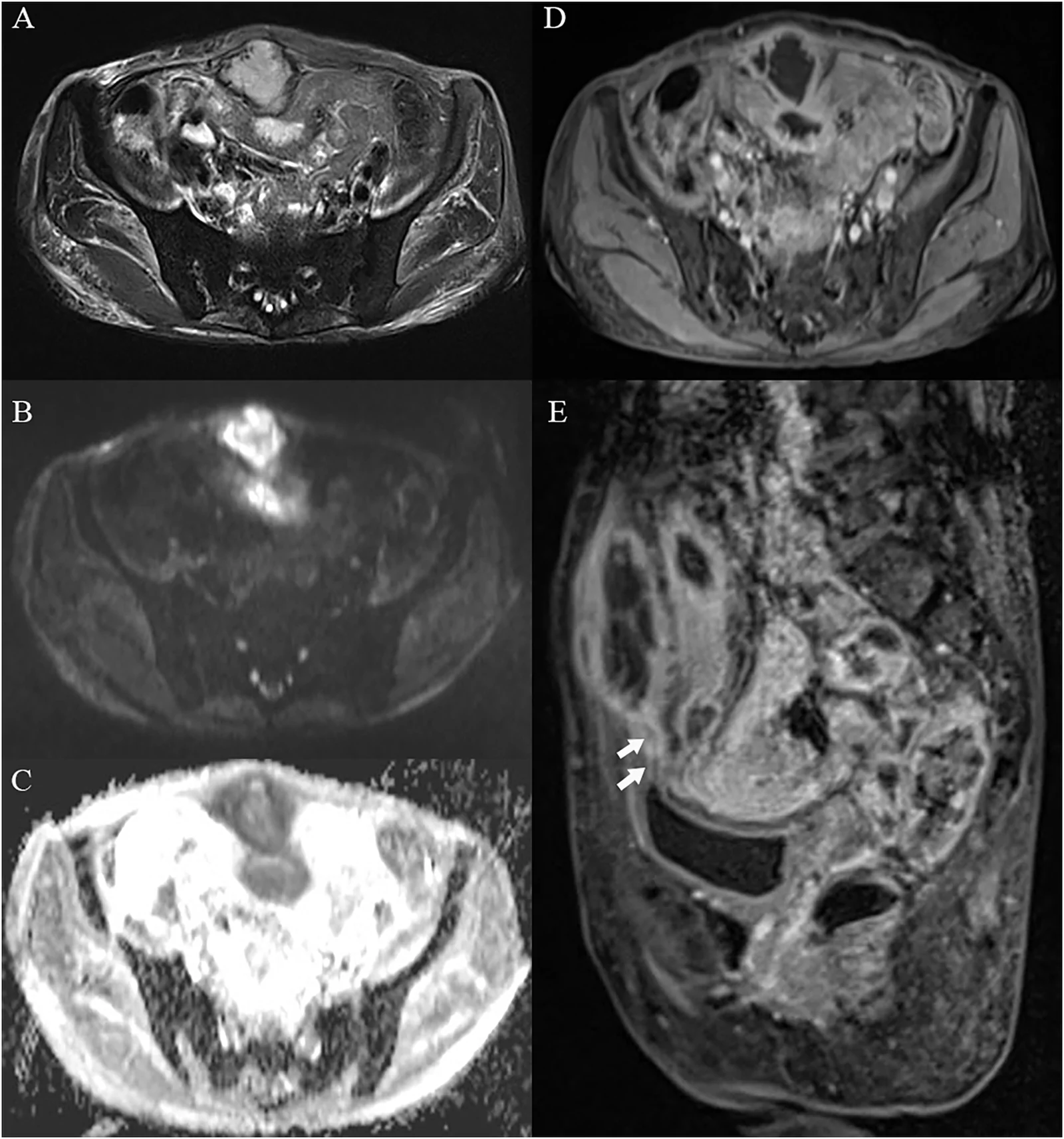
**(A)**: fat-suppressed T2-weighted imaging on the second day of admission. **(B,C)**: Diffusion-weighted imaging (b = 600) and apparent diffusion coefficient (ADC) map. **(D,E)**: Fat-suppressed contrast-enhanced T1-weighted imaging. Two adjacent thick-walled cystic lesions, one in the space of Retzius and the other posterior to the first lesion. Both lesions were hyperintense on fat-suppressed T2-weighted imaging **(A)** and diffusion-weighted imaging **(B)**, with corresponding hypointensity on the ADC map **(C)** On fat-suppressed contrast-enhanced T1-weighted imaging, both lesions showed marked peripheral rim enhancement, non-enhancing fluid content, and prominent enhancing internal septations **(D)** Sagittal contrast-enhanced T1-weighted images demonstrated continuity between the bladder and the anterior pelvic wall lesion (E, arrow).

Four days after admission, the patient underwent laparotomy. Intraoperatively, an extraperitoneal abscess cavity measuring approximately 6×5×4 cm was identified within the urachus, approximately 5 cm below the umbilicus. Ileal loops located approximately 110, 140, and 180 cm from the cecum were densely adherent to the wall of the urachal abscess, and an interloop abscess cavity had formed between these loops. A portion of the greater omentum overlaid and was adherent to the abscess cavity.

Postoperative pus culture grew Gram-negative bacilli. Histopathologic examination of the urachus revealed suppurative inflammation with abscess formation, accompanied by a focal histiocytic reaction and granulation tissue (Figures 3A, B). The patient recovered well and was discharged two weeks after admission. The timeline of the clinical course is shown in Table 1. This report follows the CARE guidelines (7).

**Figure 3:**
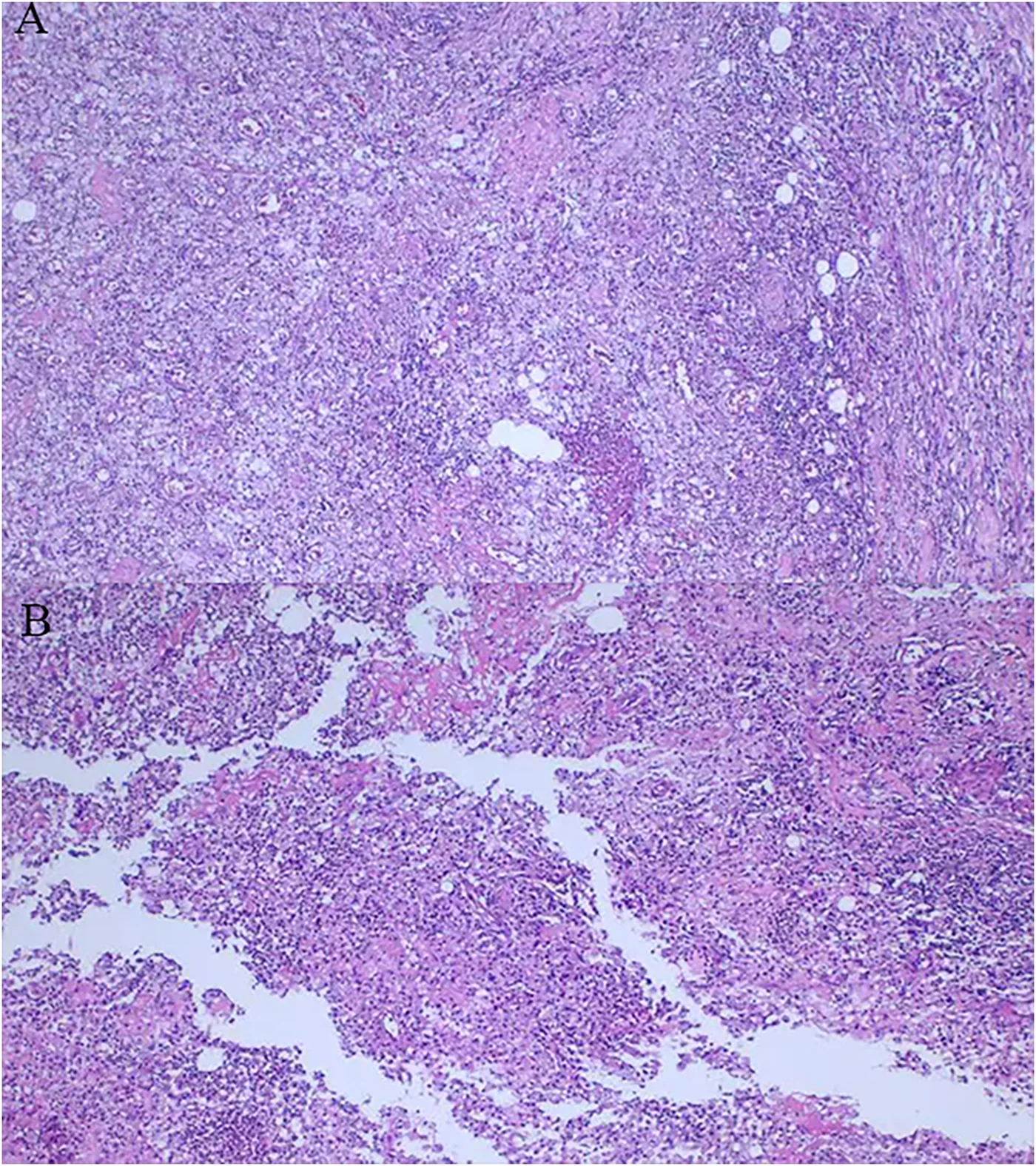
Hematoxylin and eosin staining of the urachus biopsy specimen **(A and B)**. The lesion shows an inflammatory response characterized by extensive infiltration of neutrophils, tissue necrosis, and pus formation, with histiocytic reaction and granulation tissue formation observed in the affected area.

**Table 1:** Timeline of the clinical course.

Timeline	Medical Event	Results/Notes
20 days pre-admission	Symptom onset	Paroxysmal lower abdominal pain
Admission day	Physical examination	A 7×7 cm mass in the lower abdomen
	Laboratory tests	White Blood Cell: 6.04×10^9^/L Urine leukocytes: ++ Glycated Hemoglobin: 11.54% CEA: 5.90 ng/mL CA125: 41.909 U/mL
	Computed tomography	Two thick-walled cystic lesions, peripheral rim enhancement, non-enhancing fluid content, prominent enhancing internal septations, tract-like communication
1 day after admission	Magnetic resonance imaging	Two thick-walled cystic lesions, hyperintense on diffusion-weighted imaging, peripheral rim enhancement, non-enhancing fluid content, prominent enhancing internal septations, continuous with the dome of the bladder
4 days after admission	Laparotomy	Extraperitoneal abscess within the urachus, interloop abscess between ileal loops
	Postoperative pathology	Inflammation with abscess formation, a focal histiocytic reaction, granulation tissue
2 weeks after admission	Discharge	Recovered well

## Discussion

Urachal abscess usually arises from secondary infection of a urachal cyst and classically presents with midline infraumbilical pain, fever, and a palpable mass (4, 5). However, the diagnosis is often delayed because these symptoms overlap with the nonspecific presentations of appendicitis, diverticulitis, and gynecologic disorders. Our patient presented with lower abdominal pain and an abdominal mass, but had no history of fever and no appreciable abdominal tenderness. These characteristics are similar to those reported in some previous studies and further complicate the clinical diagnosis (8, 9). We speculate that the absence of typical signs of peritonitis in this patient may be explained by the intact walls of both the urachal and interloop abscesses and by encapsulation of the inflammatory exudate within the interloop abscess by reactive fibrous tissue. In addition, because the interloop abscess lay posterior to the urachal abscess and at a greater depth, it may not have been directly palpable on physical examination, which may in turn have accounted for the absence of pronounced peritoneal irritation on palpation. Her diabetes mellitus of more than ten years' duration increased her susceptibility to infection (10). Although urinalysis revealed leukocytes (++), the patient reported no irritative bladder symptoms. Because no urine culture was obtained, the underlying cause of the leukocyturia could not be definitively determined. We speculate that this finding is attributable to the proximity of the urachal abscess to the bladder, with local inflammation leading to reactive leukocyturia. The mildly elevated serum CA125 and CEA levels were most likely reactive to intraperitoneal inflammation or caused by other factors in the patient rather than indicative of an occult neoplasm (11).

The imaging diagnosis of urachal abscess is typically based on ultrasonography (US) or CT, with MRI reserved for cases where the diagnosis remains challenging (3). US may reveal a complex cystic mass in the midline above the bladder. On contrast-enhanced CT, the lesion usually appears as a cystic mass within the space of Retzius, exhibiting a thick, irregularly enhancing wall and a central non-enhancing low-attenuation area. MRI shows a thick-walled, multiloculated mass in the space of Retzius that is hyperintense on T2-weighted imaging and displays heterogeneous enhancement after contrast administration (2, 12). In our case, non-contrast CT and MRI demonstrated a thick-walled cystic lesion in the space of Retzius, contiguous with the bladder dome and containing internal septations. Following contrast administration, both the peripheral rim and the internal septa showed marked enhancement. Furthermore, abscesses contain abundant inflammatory cells, bacteria, and necrotic debris, all of which restrict water diffusion; this manifests as hyperintensity on DWI with corresponding hypointensity on the ADC map (13). These features help differentiate a urachal abscess from a simple serous cyst, tumor necrosis, or loculated peritoneal fluid on MRI, thereby providing reliable functional imaging evidence for preoperative characterization.

Previous reports have described elderly patients in whom a large urachal abscess mimicked an intra-abdominal abscess, as well as those in whom urachal infection extended into the retroperitoneum and formed abscesses (14, 15). In these cases, however, the urachal abscess was extraperitoneal, and the secondary infection did not extend into the peritoneal cavity. To the best of our knowledge, no previous case report has described an elderly patient with both a urachal abscess and secondary interloop abscesses. A distinctive feature of this case is the coexistence of an interloop abscess with a urachal abscess. In our case, neither intraoperative findings nor imaging identified any alternative cause for the interloop abscess, such as intestinal perforation, diverticulitis, inflammatory bowel disease, or ovarian pathology. Moreover, contrast-enhanced CT clearly depicted a narrow tract-like communication connecting the two abscesses, strongly suggesting that the urachal abscess was the source. However, the tract-like communication was not identified intraoperatively, likely because its small caliber made visual detection difficult. The diagnosis of an interloop abscess between ileal loops relied primarily on intraoperative identification of an abscess cavity situated between ileal loops and overlaid by the greater omentum. Imaging demonstrated only partial encasement of the abscess cavity by bowel loops and some mesenteric exudation, and could not reliably confirm whether the posterior abscess had extended through the peritoneum from the urachal abscess and was located between bowel loops. Consequently, the final anatomical classification of this lesion depended on intraoperative findings rather than on imaging alone. Concerning the pathogenesis of the interloop abscess, we postulate that when the intraluminal pressure of the urachal abscess elevates, or inflammation erodes adjacent tissues, pus may extravasate into the peritoneal cavity. In the setting of chronic fibrous intestinal adhesions from a prior laparotomy, the spilled pus became sequestered within the mesenteric interloop spaces and was subsequently encapsulated by reactive fibrous tissue, ultimately forming a secondary interloop abscess.

In the differential diagnosis of a thick-walled cystic lesion in the space of Retzius, urachal carcinoma is the primary malignancy to consider alongside infection. Urachal carcinoma typically presents as a cystic-solid mass with heterogeneous enhancement of the solid component (16). In contrast, urachal abscesses, even when there is a septum of granulation tissue, usually show only thick and septal enhancement at the periphery, without a solid soft-tissue component. In our case, both lesions were purely cystic with rim enhancement, and no mural nodule or solid mass was identified. Combined with marked DWI hyperintensity and surrounding fat stranding, these findings helped exclude malignancy before surgery. Other differential possibilities include ovarian cystadenoma and tubo-ovarian abscess. These ovarian lesions are closely related to the adnexa. In contrast, the lesions in our case were centered anteriorly, contiguous with the bladder dome, and located in the extraperitoneal space, which permitted clear distinction on imaging.

This study has several limitations. As a single case report, the findings cannot be generalized, and the imaging features described may not be representative of all patients with urachal abscess complicated by interloop abscess. In addition, the patient's own perspective on her illness could not be obtained for inclusion, and the report therefore lacks a first-person account of the patient experience.

## Conclusion

We report a rare case of a urachal abscess complicated by a secondary interloop abscess, a combination that presented a diagnostic challenge. The patient presented with lower abdominal pain and an abdominal mass, without typical signs of peritonitis. Both demonstrated peripheral rim enhancement, enhancing internal septations, non-enhancing fluid content, and marked hyperintensity on DWI. These findings collectively were diagnostic of pyogenic abscesses. Crucially, contrast-enhanced CT depicted a tract-like communication connecting the two collections, suggesting contiguous spread from the primary urachal abscess. In this case, however, imaging had limited value in determining the location of the posterior abscess within the interloop, and the final diagnosis ultimately required surgical confirmation. Surgical treatment proved definitive and was clearly beneficial for this patient.

## Data Availability

The raw data supporting the conclusions of this article will be made available by the authors, without undue reservation.
